# Dopamine in plasma – a biomarker for myofascial TMD pain?

**DOI:** 10.1186/s10194-016-0656-3

**Published:** 2016-07-07

**Authors:** Andreas Dawson, Niclas Stensson, Bijar Ghafouri, Björn Gerdle, Thomas List, Peter Svensson, Malin Ernberg

**Affiliations:** Center for Oral Rehabilitation, Östergötland County Council, Linköping, Sweden; Department of Orofacial Pain and Jaw Function, Faculty of Odontology, Malmö University, Malmö, Sweden; Rehabilitation Medicine, Department of Medicine and Health Sciences, Faculty of Health Sciences, Linköping University and Pain and Rehabilitation Centre, Östergötland County Council, Linköping, Sweden; Occupational and Environmental Medicine, Department of Clinical and Experimental Medicine, Faculty of Health Sciences, Linköping University and Centre of Occupational and Environmental Medicine, Östergötland County Council, Linköping, Sweden; Department of Rehabilitation Medicine, Skåne University Hospital, Lund, Sweden; Section of Clinical Oral Physiology, Department of Dentistry, Aarhus University, Aarhus, Denmark; Center for Functionally Integrative Neuroscience (CFIN), MindLab, Aarhus University Hospital, Aarhus, Denmark; Section of Orofacial Pain and Jaw Function, Department of Dental Medicine, Karolinska Institutet, Huddinge, Sweden; Department of Dental Medicine, Karolinska Institutet, Huddinge, Sweden; Faculty of Odontology Malmö University, Malmö Sweden; Department of Dentistry, Aarhus University, Aarhus, Denmark

**Keywords:** Temporomandibular joint disorders, Bruxism, Dopamine, 5-HT, Masseter muscle

## Abstract

**Background:**

Dopaminergic pathways could be involved in the pathophysiology of myofascial temporomandibular disorders (M-TMD). This study investigated plasma levels of dopamine and serotonin (5-HT) in patients with M-TMD and in healthy subjects.

**Methods:**

Fifteen patients with M-TMD and 15 age- and sex-matched healthy subjects participated. The patients had received an M-TMD diagnosis according to the Research Diagnostic Criteria for TMD. Perceived mental stress, pain intensity (0–100-mm visual analogue scale), and pressure pain thresholds (PPT, kPa) over the masseter muscles were assessed; a venous blood sample was taken.

**Results:**

Dopamine in plasma differed significantly between patients with M-TMD (4.98 ± 2.55 nM) and healthy controls (2.73 ± 1.24 nM; *P* < 0.01). No significant difference in plasma 5-HT was observed between the groups (*P* = 0.75). Patients reported significantly higher pain intensities (*P* < 0.001) and had lower PPTs (*P* < 0.01) compared with the healthy controls. Importantly, dopamine in plasma correlated significantly with present pain intensity (*r* = 0.53, *n* = 14, *P* < 0.05) and perceived mental stress (*r* = 0.34, *n* = 28, *P* < 0.05).

**Conclusions:**

The results suggest that peripheral dopamine might be involved in modulating peripheral pain. This finding, in addition to reports in other studies, suggests that dopaminergic pathways could be implicated in the pathophysiology of M-TMD but also in other chronic pain conditions. More research is warranted to elucidate the role of peripheral dopamine in the pathophysiology of chronic pain.

## Background

Dopamine and 5-hydroxytryptamine (5-HT) are neurotransmitters found in the central (CNS) and peripheral nervous systems, and in plasma. In the CNS, dopamine is involved in motor control, cognition, and the reward system [1], but possibly also in pain perception [2]. Primarily dopaminergic neurons synthesize dopamine in the CNS [1], while neuronal fibers, adrenal medulla, and neuroendocrine cells synthesize dopamine in the peripheral nervous system [1]. Goldstein and Holmes have observed that dopamine in plasma is primarily related to sympathetic activity; that is, rises in sympathetic activity increase release of precursors to dopamine and norepinephrine [3]. Rubi and Maechler report in their mini-review that dopamine in plasma are elevated in situations that involve psychological stress, muscle exercise, or hypovolemia [1].

Bruxism, “a repetitive jaw-muscle activity characterized by clenching or grinding of the teeth and/or by bracing or thrusting the mandible” [4], has been linked to disturbances in the central dopaminergic system [5], as has another motor function disorder, Parkinson’s disease [6]. Aside from motor function-related disturbances, the most self-reported symptom in patients with Parkinson’s disease is pain [7]. It has also been suggested that alterations in dopaminergic neurotransmission might be involved in burning mouth syndrome [8, 9], fibromyalgia [10], and chronic orofacial pain [8, 9]. The literature indicates that the dopaminergic system may be involved in central pain modulation, but the role of dopamine at the peripheral level in relation to pain is unknown.

Graven-Nielsen et al. found 5-HT involvement in pain transmission at the peripheral and central levels [11]. Several other studies have demonstrated that patients with chronic myalgia, including myofascial temporomandibular disorders (M-TMD) have significantly higher interstitial concentrations of 5-HT than healthy controls [12–16] and that high 5-HT levels are correlated with muscle pain and allodynia [14–17]. In addition, in patients with fibromyalgia, Ernberg et al. found that high levels of plasma 5-HT in relation to serum 5-HT may be associated with pain discomfort and high levels of anxiety [18].

Amongst others, autonomic and psychosocial factors have been suggested as risk factors for TMD [19, 20]. Maixner et al. observed that TMD patients have a significantly altered autonomic function compared with healthy controls at rest and in stressful situations [20]. Thus, it is plausible that activity in the sympathetic nervous system of these patients is altered, and if dopamine in plasma is a potential indicator of such activity, they could be implicated in M-TMD.

The role of peripheral dopamine and 5-HT and their association with pain in patients with M-TMD is so far unknown. The aim of this study was to investigate plasma levels of dopamine and 5-HT in patients with M-TMD and healthy controls. Two hypotheses were tested: *(i)* patients with M-TMD have higher levels of dopamine and 5-HT in plasma than healthy subjects, and *(ii)* pain intensity and the pressure pain threshold correlate with plasma levels of dopamine and 5-HT in patients with M-TMD.

## Methods

### Participants

Fifteen patients with M-TMD (mean age: 31.8 ± 13.4 years) and an age- and gender-matched control group (mean age: 31.6 ± 12.4 years) participated in this study. Eleven females and four males participated in each group. Both groups were participating in other studies [12, 21, 22]. The control group was recruited from staff at Malmö University, while the patients were recruited among consecutive patients referred to the Department of Orofacial Pain and Jaw Function, Faculty of Odontology, Malmö University, Malmö, Sweden and, after a clinical examination, had been diagnosed with M-TMD [23]. One author (AD) examined the patients to confirm a diagnosis of M-TMD and the control group to confirm that the subjects were healthy.

The inclusion criteria for the M-TMD group were *(i)* age > 18 years, *(ii)* a diagnosis of M-TMD pain [23], *(iii)* moderate or more severe pain in the masseter muscles upon palpation at the clinical examination and on the experimental day, and *(iv)* continuous or persistent pain in the jaw muscles for more than 6 months.

Inclusion criteria for the healthy controls were *(i)* age > 18 years, *(ii)* healthy, and *(iii)* no orofacial pain complaints.

Exclusion criteria for both groups were conditions that could influence pain sensitivity, such as systemic inflammatory connective tissue diseases (e.g., rheumatoid arthritis); whiplash-associated disorders; chronic widespread muscle pain conditions (e.g., fibromyalgia); neuropathic pain or neurological disorders (e.g., oromandibular dystonia); pain of dental origin; pregnancy or lactation; high blood pressure; and ongoing dental treatment. Another exclusion criterion was medication that could interfere with the blood analysis or pain sensitivity (e.g., anticoagulant treatment and analgesic consumption within 1 week before the experiment, for example, of paracetamol, NSAIDs, salicylate drugs, or opioids) or that could influence pain perception (e.g., anti-depressants or anti-epileptic drugs). Exclusion criteria were assessed in each subject by taking a medical history.

### Ethics approval of the study

This study was conducted at the Department of Orofacial Pain and Jaw Function at Malmö University. The guidelines of the Helsinki Declaration were followed, and the Regional Ethics Review Board at Lund University approved the study (2010/31). Before participation, subjects signed an informed-consent form and were informed that they could refrain from the study at any time without any consequences. After completion of participation, subjects were financially compensated.

### Study design

The 1-h clinical examination, scheduled at 8 am or 1 pm comprised an examination according to the RDC/TMD; collection of a venous blood sample for analysis of dopamine and 5-HT in plasma, and assessment of present pain intensity and the pressure pain threshold (PPT). Throughout the trial, subjects sat upright in a dental chair with head support. One operator (AD) conducted the examination.

### Blood sampling

Venous blood samples were collected in 3-mL EDTA Vacutainers (Becton, Dickinson, and Company; Franklin Lake, NJ, USA). Directly after withdrawal, the samples were centrifuged (2000 g, +20 °C) for 10 min and the plasma was transferred into Eppendorf tubes and stored at −70 °C until analysis.

### Assessment of pressure pain threshold, present pain intensity, and perceived mental stress

An electronic algometer (Somedic Sales AB, Hörby, Sweden) was used to assess the PPT, defined as the amount of pressure (kPa) needed to produce the slightest sensation of pain. A 1-cm^2^ probe was applied to the lower attachment of the right masseter muscle with a constant pressure of 30 kPa/s. The mean of three measurements made at 60-s intervals at the same area was calculated [24]. The reliability of PPT measurements on the masseter muscle was previously found to be acceptable [25].

A 100-mm visual analogue scale (VAS) was used to assess pain intensity (anchor definitions: no pain and worst imaginable pain).

The 10-item perceived stress scale (PSS-10) was used to assess perceived mental stress during the past month in the participants. The PSS-10 measures to what degree various situations in life are perceived as stressful. Reliability and validity of the Swedish version of PSS-10 is good [26].

### Analysis of dopamine and 5-HT

On the day of analysis, the plasma samples were thawed on ice. Evolute WCW solid phase extraction columns (25 mg) from Biotage (Uppsala, Sweden) were used to extract dopamine and 5-HT from the plasma. The samples were prepared using the manufacturer’s recommended extraction protocol. After the analytes were eluted from the columns, the fractions were dried using a SpeedVac system and dissolved in the mobile phase used for high performance liquid chromatography with electrochemical detection (HPLC-EC). HPLC analyses were conducted as previously described [13].

### Statistics

All statistical analyses were done using the Statistical Package for the Social Sciences for Windows (SPSS, v.20; IBM). Analyses were performed two-tailed at a significance level of 5 %. All variables are expressed as means and SDs unless otherwise stated. The Shapiro-Wilk’s test was used to test the data for normality. Present pain intensity, PPT, perceived mental stress, and the levels of dopamine in plasma were normally distributed, while 5-HT levels in plasma were normally distributed after Ln-transformation. Parametric statistics were used in all statistical analyses excluding those of categorical variables or in analysis of M-TMD duration, since this variable was not normally distributed.

The Mann-Whitney *U*-test was used to determine significant differences between groups regarding duration of M-TMD (mos) and number of muscle and temporomandibular joints sites with pain on palpation.

Independent samples *t*-tests were used to investigate significant differences in age, pain intensity, PPT, perceived mental stress, and dopamine and 5-HT in plasma between groups. To test for significant correlations between pain intensity, PPT, perceived mental stress, and plasma dopamine, or 5-HT in each group, the Pearson’s correlation test (adjusted for multiple testing with Bonferroni correction) was used.

It has been suggested that plasma levels of dopamine and epinephrine are equivalent [1], so the sample size calculation was based on the mean ± SD of the plasma level of epinephrine in healthy subjects, 0.337 ± 0.106 nM [27], using β = 0.80 and α = 0.05. A power analysis found that 11 subjects in each group would suffice to detect a mean difference of 1 SD in dopamine level.

## Results

### Clinical characteristics of the study sample

Table 1 presents the clinical characteristics of the study sample.

**Table 1:** Clinical characteristics of the study sample

	Patients with M-TMD	Controls	*P*	Test
Subjects				
All	15	15		
Females	11	11		
Males	4	4		
Age (yrs)	31.8 ± 13.4	31.6 ± 12.4	0.966	Independent samples *t*-test
Duration of M-TMD (months)	58.6 ± 60.3	0.0 ± 0.0	<0.001	Mann-Whitney U test
No. of muscle sites with pain on palpation (max. 20)	14 (8)	2 (2)	<0.001	Mann-Whitney U test
No. of TMJ sites with pain on palpation (max. 4)	1 (1)	0 (0)	<0.001	Mann-Whitney U test
Perceived mental stress (PSS-10)	16.6 ± 5.9	12.1 ± 3.6	<0.05	Independent samples *t*-test
Self-reported awake bruxism	11	3		
Self-reported sleep bruxism	11	7		
Blood sampling at 8 am	8	8		
Blood sampling at 1 pm	7	7		

Mean ± SD, age (yrs), duration of M-TMD (mos), and perceived mental stress; median (interquartile range) number of muscle and temporomandibular joint (TMJ) sites with pain on palpation; frequency of self-reported awake and sleep bruxism, and blood sampling time, in 15 patients with M-TMD and 15 healthy subjects

### Dopamine and 5-HT in plasma

**Fig. 1:**
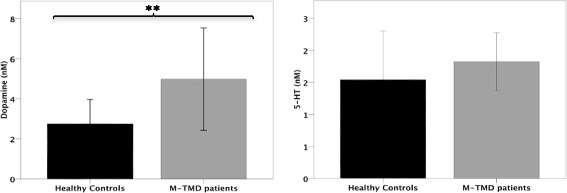
Mean ± SD plasma concentration of dopamine and serotonin (5-HT) in 15 patients with M-TMD and 15 healthy controls. Significant differences between groups, **P < 0.01

Dopamine in plasma differed significantly between groups (*P* < 0.01), being higher for the patients with M-TMD than the healthy subjects. No significant group differences for plasma 5-HT were observed (*P* = 0.75; Fig. 1).

### Present pain intensity, pressure pain threshold, and perceived mental stress

**Fig. 2:**
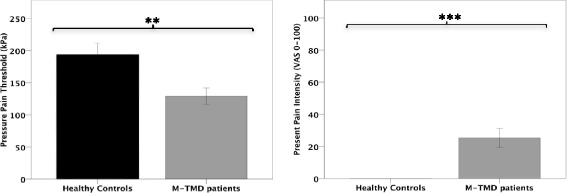
Mean ± SD pressure pain threshold (kPa), and present pain intensity (VAS 0–100) assessed in 15 patients with M-TMD and 15 healthy controls. Significant differences between groups, **P < 0.01; ***P < 0.001

**Fig. 3:**
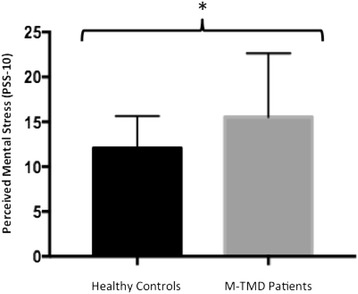
Mean ± SD perceived mental stress assessed in 15 patients with M-TMD and 15 healthy controls. Significant differences between groups, * *P* < 0.05

Patients with M-TMD had significantly higher present pain intensity (*P* < 0.001; Fig. 2) and perceived mental stress (*P* < 0.05; Fig. 3) and significantly lower PPTs compared with healthy subjects (*P* < 0.01; Fig. 2).

### Correlations

In the M-TMD group, present pain intensity correlated significantly with dopamine in plasma (*r* = 0.53, *n* = 14, *P* < 0.05), but not with 5-HT (*r* = −0.02, *n* = 14, *P* > 0.05). A significant correlation was observed between dopamine in plasma and perceived mental stress for the group as a whole (*r* = 0.34, *n* = 28, *P* < 0.05). Present pain intensity correlated significantly with PPT (*r* = −0.42, *n* = 30, *P* < 0.01) and perceived mental stress (*r* = 0.46, *n* = 29, *P* < 0.01). 5-HT did not correlate significantly with present pain intensity (*r* = 0.005, *n* = 27, *P* > 0.05), PPT (*r* = −0.011, *n* = 27, *P* > 0.05), or perceived mental stress (*r* = 0.104, *n* = 27, *P* > 0.05).

## Discussion

The main findings of this study were that *(i)* dopamine in plasma was significantly higher in patients with M-TMD than in healthy subjects; and correlated significantly with present pain intensity and perceived mental stress, (*ii*) present pain intensity correlated significantly with PPT and perceived mental stress, (*iii*) 5-HT in plasma did not differ significantly between groups, and did not correlate significantly with present pain intensity, PPT, or perceived mental stress.

Previous studies have suggested that dopaminergic neurotransmission is altered at a central level in patients with chronic pain conditions [8–10]. But the novel finding in this study that dopamine in plasma correlated significantly with present pain intensity in the masseter muscle suggests involvement of dopamine in pain modulation, at not only a central but a peripheral level as well. It is known that dopamine targets D1 and D2 receptors at the central level. Activation of the D1 receptor leads to formation of cAMP [28] and subsequent activation of protein kinase A, which sensitizes tetrodotoxin (TTX)-resistant Na^+^ channels [29], causing a pain response. On the other hand, activation of D2 receptors counteracts cAMP formation [28], possibly inhibiting sensitization of TTX-resistant Na + channels [29]. Such inhibition has been observed in animal studies where administration of D2 dopamine receptor antagonists in striatal neurons reduces pain [30, 31]. On the other hand, however, it is believed that patients with chronic pain, such as burning mouth syndrome and atypical facial pain, exhibit higher densities of D2 receptors in the putamen compared with healthy subjects [8, 9]. Thus, it seems that D2 receptors have both a pain-suppressing and a pain-enhancing effect. Hypothetically, chronic activity in the dopaminergic system, possibly due to chronic pain, could cause these effects; in healthy individuals, the dopaminergic system is not chronically activated, so D2 receptors seem to be primarily associated with pain suppression.

An experimental animal study of Tambeli et al. demonstrated the existence of D1 receptors in the periphery [32]. The researchers [32] showed that dopamine injections provoked a nociceptive response, while the selective D1-receptor antagonist SCH23390 reversed this response. Similarly, 5-HT injections also created a nociceptive response that was reversed by the same D1-receptor antagonist [32]. Thus, it seems that 5-HT in the periphery provokes a release of dopamine, which in turn targets D1 receptors, thus causing a nociceptive response. This is particularly interesting since patients with M-TMD have significantly higher levels of interstitial 5-HT than healthy controls [14–16, 22]. Thus, human D1 receptors could possibly be activated by the same indirect mechanism observed by Tambeli et al. [32], contributing to the facilitation of pain. In other words, it is possible that dopamine is involved in the pathophysiology of M-TMD at a peripheral level, especially since dopamine in plasma was significantly correlated with present pain intensity.

The patients with M-TMD reported sleep bruxism and awake bruxism more often than the healthy subjects. One thought is that these bruxism habits influenced the results of the present study, since bruxism has been linked to the central dopaminergic system [5]. This is unlikely, however, since central dopamine do not pass the blood-brain barrier and is therefore differed from peripheral dopamine [1].

Circadian variation in plasma dopamine levels has been reported in humans [33], and could influence the results. In the present study, equal numbers of patients with M-TMD and healthy subjects were scheduled at 8 am and 1 pm. The influence of circadian variation on plasma dopamine levels in this study should thus be minimal.

The hypothesis that patients with M-TMD would have significantly higher levels of 5-HT in plasma than healthy subjects was not confirmed. Ernberg et al. showed that a high level of 5-HT in plasma relative to in serum was significantly correlated with pain in patients with fibromyalgia [18] and that serum levels of 5-HT were significantly correlated with allodynia in patients with M-TMD [14–16]. It must be emphasized that blood plasma analyses measure the free fraction of 5-HT, while serum analyses reflect the total amount of 5-HT. Patients with M-TMD have higher levels of interstitial 5-HT in the masseter muscles compared with healthy controls [22], thus it was hypothesized that the free fraction of 5-HT (plasma) would be significantly higher in these patients, due to an increased release from the thrombocytes, but this was not confirmed and needs to be further investigated. One explanation for these conflicting results may be that the patients with M-TMD in our study had localized pain in the masticatory muscles, while in the Ernberg et al. study [18], the patients had wide spread pain, that is, fibromyalgia.

In the present study, patients with M-TMD had significantly higher levels of perceived mental stress than healthy controls, which is in line with findings from other studies [34, 35]. We also found that perceived mental stress correlated significantly with present pain intensity and dopamine in plasma. A likely possibility is that M-TMD pain is a potential stressor. This may lead to mental stress and activation of the sympathetic nervous system (i.e., two factors that are reported to be risk factors for M-TMD pain [20, 36] with subsequent peripheral release of dopamine. Measuring heart rate variability is one way to assess sympathetic activity [37], but this was not done in the present study. On the other hand, dopamine in plasma is mainly regulated by activity in the sympathetic nerves [3]. The results of the present study showed a significant correlation between dopamine in plasma and perceived mental stress, which supports the suggestion that peripheral dopamine is mainly regulated by sympathetic activity [3].

We observed that the patients with M-TMD had significantly lower PPTs in the masseter muscle than healthy controls, which is in line with the results of several other studies (e.g., [38, 39]. This further confirms the generally accepted knowledge that, apart from central mechanisms, peripheral mechanisms are involved in the pathophysiology of M-TMD [40]. 5-HT is involved in peripheral sensitization, and the level of interstitial 5-HT is significantly increased in M-TMD patients compared with healthy controls [14–16, 22]. It is believed that 5-HT may provoke a peripheral dopamine release [32] that can facilitate a pain response by sensitizing tetrodotoxin (TTX)-resistant Na^+^ channels [29]. Therefore, it is possible that peripheral dopamine contributes to peripheral sensitization, and could be an explanation for the lower PPTs observed in M-TMD patients compared with the healthy controls.

A limitation of the study is that the participants not were asked about present perceived mental stress in the experimental session. Sex hormones can alter pain levels [41], and a limitation is that female participants were not queried about menstrual cycle phase. It is, however, very likely that the females were in different phases of the menstrual cycle, which would negate any effects. A strength of this study is that the present findings are the first to highlight that peripheral dopamine could be involved in peripheral pain modulation.

## Conclusions

In conclusion, the novel finding of the present study that peripheral dopamine may be involved in peripheral pain modulation means (indicates) that peripheral dopamine could be implicated in the pathophysiology of M-TMD and other chronic pain conditions. But, whether the increased level of peripheral dopamine is a consequence of pain or mental stress is not known. More research is needed to further understand the role and mechanisms of peripheral dopamine in chronic pain and mental stress.
